# MEK targeting in N-RAS mutated metastatic melanoma

**DOI:** 10.1186/1476-4598-13-45

**Published:** 2014-03-04

**Authors:** Jaykumar Thumar, David Shahbazian, Saadia A Aziz, Lucia B Jilaveanu, Harriet M Kluger

**Affiliations:** 1 Section of Medical Oncology, Yale Cancer Center, Yale School of Medicine, 333 Cedar Street, WWW213, New Haven, CT 06520, USA

**Keywords:** Targeted therapy, Melanoma, N-RAS, MEK inhibitor, Apoptosis

## Abstract

**Background:**

Gain of function mutations in B-RAF and N-RAS occur frequently in melanoma, leading to mitogen activating protein kinase (MAPK) pathway activation, and this pathway is the target of drugs in development. Our purpose was to study clinical characteristics of patients with mutations in this pathway and to determine activity of inhibitors of B-RAF and MEK in short term cultures grown from tumors of some of these patients.

**Methods:**

Clinical and pathologic data were collected retrospectively on melanoma patients tested for B-RAF and N-RAS mutations at the Yale Cancer Center and associations with survival were determined. We studied *in vitro* activity of the pan-RAF inhibitor, RAF265, and the MEK inhibitor, MEK162, in 22 melanoma short term cultures. We further characterized the effect of MEK inhibition on apoptosis and growth of melanoma cultures.

**Results:**

In a cohort of 144 metastatic melanoma patients we found that patients with N-RAS mutant melanoma had a worse prognosis. These patients were more likely to have brain metastases at the time of presentation with metastatic disease than their N-RAS-wild-type counterparts. All N-RAS mutant melanoma cultures tested in our study (n = 7) were sensitive to MEK inhibition162. Exposure to MEK162 reduced ERK1/2 phosphorylation, and induced apoptosis. Clonogenic survival was significantly reduced in sensitive melanoma cell cultures.

**Conclusions:**

The prognosis of patients with melanoma expressing constitutively active N-RAS is poor, consistent with studies performed at other institutions. N-RAS mutant melanoma cultures appear to be particularly sensitive to MEK162, supporting ongoing clinical trials with MEK162 in N-RAS mutated melanoma.

## Background

Melanoma is the leading cause of fatal skin cancer, and in recent years, the incidence and mortality of melanoma have increased. Prior to the recent advances in therapy for patients with stage IV disease, the prognosis of metastatic melanoma was very poor with a median survival of less than 12 months [1]. One of the most significant advances in recent years was the elucidation of the etiological role of the mitogen activated protein kinase (MAPK) pathway in melanomagenesis, particularly the roles of mutant B-RAF and N-RAS [2]. The RAS-RAF-MAPK signaling pathway is activated in the vast majority of melanomas, either due to increased growth factor signaling or by genetic alterations in N-RAS and B-RAF proteins [3]. Thus, the MAPK pathway is a key therapeutic target, and activation of this pathway has prognostic importance in melanoma as well [4].

Mutations in B-RAF or N-RAS are found in the majority of melanomas, and are often identified in benign nevi as well [5,6]. Activating mutations in B-RAF and N-RAS occur in 40-60% and 15-25% of melanomas, respectively. Several recent studies have examined the associations between B-RAF and N-RAS mutations and clinical characteristics and prognosis in patients with metastatic melanoma [4,7,8]. Patients with N-RAS and B-RAF mutations have a higher incidence of CNS (central nervous system) metastasis at the time of diagnosis of stage IV disease compared to patients who are wild-type for B-RAF and N-RAS, and N-RAS mutation status was identified as an independent predictor of shorter survival after a diagnosis of stage IV melanoma [9].

While the precise role of B-RAF mutations in oncogenesis is unclear, such mutations result in the constitutive activation of the MAPK pathway and enhanced growth and vascular development in melanoma tumors [10]. Similarly, mutations in N-RAS cause activation of downstream serine/threonine kinases (including B-RAF and PI3K), which promote cell cycle progression, cellular transformation, and enhanced cell survival [11]. B-RAF is an important therapeutic target, and inhibition of mutant B-RAF has resulted in antitumor activity and improved survival in patients with metastatic melanoma expressing constitutively active B-RAF^V600E^[12]. Vemurafenib (Roche-Genentech), an inhibitor of B-RAF kinase with increased selectivity for mutant B-RAF^V600E^, was approved in 2011 by the Food and Drug Administration for treatment of unresectable melanoma harboring B-RAF^V600^ mutations [13]. Dabrafenib (GSK2118436, GlaxoSmithKline Pharmaceuticals), another specific inhibitor of mutant B-RAF^V600^ kinase, was approved for this indication in 2013, as was Trametinib (GSK1120212, GlaxoSmithKline Pharmaceuticals), an orally available and selective inhibitor of MEK1/2 [14,15]. These three were approved based on improved overall survival compared to chemotherapy in phase III clinical trials. Thus, targeting mutant B-RAF and downstream pathway members has significantly changed the management of B-RAF mutant metastatic melanoma.

RAS protein isoforms are the immediate upstream regulators of B-RAF and constitutively activating mutations in N-RAS are found in 15-25% of metastatic melanomas. RAS isoforms function as molecular switches in signal transduction cascades [16]. RAS GTPases activate their downstream effectors when bound to GTP and become inactivated once they hydrolyze GTP to GDP [17]. Being catalytically inefficient, this biochemical reaction requires co-factors, such as GTPase activating proteins (GAPs). Members of another group of enzymes, GTP exchange factors (GEFs), are necessary to re-activate RAS by promoting the release of RAS-bound GDP [18]. GTP then competes with GDP for RAS binding. Constitutively active mutant RAS molecules lose the ability to hydrolyse GTP, even in presence of GAPs [19]. Mutated RAS isoforms are found in 33% of all cancers [16]. Nevertheless, attempts to develop molecules that target biological activity of mutant RAS directly have, so far, been unsuccessful. For instance, attempts have been made to inhibit RAS using farnesyltransferase inhibitors [20]. Farnesylation is a post translational modification that enables RAS proteins to attach to the cellular membrane, where they meet their upstream and downstream signaling partners [21]. Farnesyltransferase is responsible for transferring a farnesyl group from farnesyl pyrophosphate to the pre-RAS protein. However, use of farneslytransferase inhibitors in clinical trials yielded disappointing results [22]. Strategies indirectly modulating the activity of RAS through inhibition of RAS-GEFs, stimulation of RAS-GAPs and targeted sensitization of oncogenic RAS to physiological GAP activity have been proposed (reviewed in [18]). Although B-RAF inhibitors could be hypothetically used in N-RAS mutated melanoma to target the pathway downstream of N-RAS, vemurafenib causes paradoxical hyperactivation of MEK–ERK1/2 signaling, activates C-RAF, and promotes growth in mutant N-RAS cell lines [23,24]. Thus, alternative targets are needed to inhibit growth of tumors with N-RAS mutations.

MEK1/2 are members of the RAS/RAF/MEK/ERK signaling pathway, and inhibition of MEK might result in decreased pathway activation in N-RAS and B-RAF mutant melanomas. A recent report identified new mutations in N-RAS and MEK as escape mechanisms through which B-RAF mutant melanomas acquire resistance to B-RAF inhibitors [25]. Combined treatment with dabrafenib and trametinib was able to overcome resistance in preclinical models and use in patients with B-RAF mutated tumors resulted in improved progression free survival [26].

To verify the clinical significance of B-RAF and N-RAS mutations in our institutional patient cohort we performed a retrospective analysis of patients with advanced melanoma who underwent treatment at the Yale Cancer Center and for whom sequencing for both B-RAF (exon 15) and N-RAS (exons 1 and 2) mutations was done. Furthermore, we studied the pre-clinical activity of a pan-RAF inhibitor, RAF265 (Novartis Pharmaceuticals, Basel, Switzerland), and a MEK1/2 inhibitor MEK162 (Novartis) on a panel of 22 early passage, patient-derived melanoma cell cultures. We characterized the effect of MEK162 on melanoma cell proliferation, clonogenicity and apoptosis.

## Results

### Clinical profiles of patients whose tumors harbor N-RAS and B-RAF mutations

Characteristics of our cohort of 144 patients with stage IV melanoma are shown in Table 1. Mutations were found in B-RAF in 43.7%, N-RAS in 27.7%, and 28.4% were wild type (WT) for both. The majority of B-RAF mutations were represented by substitution of valine at position 600 to glutamic acid (74.6% were B-RAF^V600E^) or to lysine (19% were B-RAF^V600K^). Substitutions of glutamine 61 accounted for 95% of N-RAS mutations (most frequently Q61R/K/L/H). The slightly higher percentage of N-RAS mutant melanomas in our population than what is commonly reported may be a result of the relatively small sample size or a reflection of local demographics.

**Table 1:** Clinical and pathological characteristics

**Clinical characteristics**		**B-RAF mutated**		**N-RAS mutated**		**Wild type**	**P value**
**N = 144**		**N = 63 (43.7)**		**N = 40 (27.7)**		**N = 41 (28.4)**	
Frequency of mutations		V600E	47(74.6)	Q61R	16 (40)		
		V600K	12 (19)	Q61K	12 (30)		
		V600R/L	2 (3.1)	Q61L	6 (15)		
		Unknown	2 (3.1)	Q61H	4 (10)		
				G12D/V	2 (5)		
Sex	Male	42 (66)		25 (62)		22 (54)	0.21
	Female	21 (33)		15 (38)		19 (46)	
Median age (years)		57.6		68.2		66.3	<0.0001
LDH	Elevated	28		19		20	0.6
	Normal	24		6		12	
	Unknown	11		15		9	
M stage	M1a	13		8		9	0.3
	M1b	11		14		9	
	M1c	39		18		23	
Soft tissue and skin metastasis		23 (37)		28 (70)		17 (41)	0.0025
Lymph node metastasis		29 (46)		30 (75)		25 (61)	0.01
Lung metastasis		41 (65)		24 (60)		27 (66)	0.8
Liver metastasis		25 (39)		11 (27)		17 (41)	0.3
Bone metastasis		17		7		6	0.3
CNS metastasis		31 (49)		16 (40)		16 (39)	0.5
Median survival from diagnosis of stage IV disease		18.3		13.0		19.6	0.17

**Figure 1:**
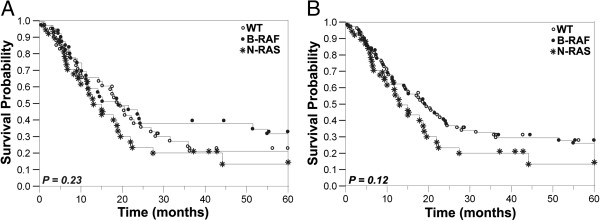
**Correlation between N-RAS mutation status and survival probability in melanoma patients.** Kaplan-Meier survival curves demonstrating the overall survival from time of diagnosis of stage IV disease. Panel **A** shows survival curves for all three patient groups, whereas panel **B** shows the patients with N-RAS mutations compared to B-RAF and WT combined. Patients with N-RAS mutations had a trend towards shorter median survival when compared to the non N-RAS WT counterparts (p = 0.12).

Patients with B-RAF mutations tended to be younger; median age at initial diagnosis of melanoma was 57.6 in patients with B-RAF mutations, 68.2 in patients with N-RAS mutations and 66.3 years in patients wild-type for both (P <0.0001). Our cohort included 22 patients who received either dabrafenib (N = 1), vemurafenib (N = 19), a pan-RAF inhibitor (N = 1) or a pan-RAF inhibitor and vemurafenib (N = 1). Patients with N-RAS mutated melanomas appear to have an increased rate of skin and soft tissue involvement (70%) compared to B-RAF mutated counterparts and WT patients (37% and 41% respectively, P = 0.0025). The rate of lymph node metastasis was also noted to be higher in patients with N-RAS mutations (75%) compared to B-RAF mutant and WT patients (46% and 61%, respectively) (P = 0.01). No statistically significant difference was seen between the genotypes and other clinical characteristics, such as M stage and LDH levels.Seeing that B-RAF inhibitors can affect survival in patients with B-RAF mutant melanomas, this group of patients was removed from the survival analysis. By Cox univariate analysis we found a trend towards shorter survival in the N-RAS mutant population, compared to the B-RAF and WT groups combined (p = 0.12). The median survival was 13 and 19.6 months, respectively. Kaplan-Meier survival curves are shown in Figure 1a for the three groups of patients (BRAF or NRAS mutant or WT for both) and Figure 1b for the two groups (NRAS compared to BRAF mutant and WT combined) to visually demonstrate the differences between the groups.

Interestingly, analysis of anatomic sites at the time of initial diagnosis of stage IV disease revealed a higher rate of brain involvement among B-RAF (16%) and N-RAS (15%) mutant melanoma patients, compared with patients with WT disease (2.5%) (P =0.04). With longitudinal follow-up, however, the rate of development of brain metastases did not differ among the three groups, possibly because the WT groups lived longer and thus developed brain metastases over time.

### *In vitro* activity of B-RAF and MEK inhibitors in a large panel of melanoma cultures

To investigate the effect of B-RAF and MEK inhibition in melanoma cultures, we used RAF265 (a pan-RAF inhibitor), MEK162 (a MEK1/2 inhibitor) and the MEK inhibitor trametinib. A panel of 22 patient-derived melanoma cultures was used; the IC_50_ for RAF265 and MEK162 are shown in Table 2. This was compared to the IC_50_ for trametinib (Additional file 1: Table S1).

**Table 2:** Patient-derived melanoma cultures with their B-RAF/N-RAS mutational status and sensitivity to RAF265 and MEK162

	**Name of the culture**	**Mutation**	**RAF265 IC**_**50**_**s [nM]**	**MEK162 IC**_**50**_**s [nM]**
**Wild type** **B-RAF /N-RAS**	YUHOIN	WT	>1000	>1000
	YUROB	WT	25	10
	YUROL	WT	>1000	>1000
	YUSOC	WT	72	36
	YUVON	WT	>1000	>1000
**B-RAF mutants**	YUCOT	V600E (GAG/GAG)	35	<1
	YUGEN	V600E (GAG/GAG)	5	108
	YUKOLI	V600E/WT (GAG/GTG)	18	27
	YUKSI	V600K (AAG/AAG)	>1000	150
	YUMAC	V600K (AAG/AAG)	208	8
	YURIF	V600K (AAG/AAG)	412	45
	YUSAC	V600E (GAG/GAG)	>1000	148
	YUSIT	V600K/WT (AAG/GTG)	188	25
	YUSUBA	V600E (GAG/GAG)	660	50
	YUZEAL	V600R (AGG/AGG)	124	33
**N-RAS mutants**	YUCHER	Q61R (CGA/CGA)	70	6
	YUDOSO	Q61K/WT (AAA/CAA)	62	9
	YUFIC	Q61R/WT (CGA/CAA)	351	5
	YUGANK	Q61K (AAA/AAA)	>1000	5
	YUGASP	Q61L (CTA/CTA)	>1000	10
	YUKIM	Q61R (AGA/AGA)	559	8
	YUTICA	Q61R/WT (CGA/CAA)	371	13

Cells were treated with each drug individually at concentrations ranging from 1 nM to 1000 nM and analyzed three days later. As shown in Table 2, the IC_50_ for RAF265 ranged from 24 to >10000 nM, 4 to 2004 nM, and 62 to 2082 nM for WT, B-RAF mutant and N-RAS mutant cultures, respectively. The IC_50_ for MEK162 ranged from 10 to >10000 nM, < 1 to 150 nM, and 4 to 13 nM for WT, B-RAF mutant and N-RAS mutant melanoma cultures, respectively.

The sensitivity to RAF265 in wild type (2 out of 5) and N-RAS (2 out of 7) melanoma cultures was low. Two wild type cultures (YUROB and YUSOC) are sensitive to both RAF265 and MEK162. Six of ten B-RAF mutant cultures were sensitive to RAF265, and seven out of ten were sensitive to MEK162. In N-RAS mutant melanoma cultures, 2 out of 7 were sensitive to RAF265 and, strikingly, all were sensitive to MEK162. Of the 7 N-RAS mutant cultures, 5 were sensitive to trametinib. YUFIC and YUTICA were more resistant.

### Molecular effects of MEK162

**Figure 2:**
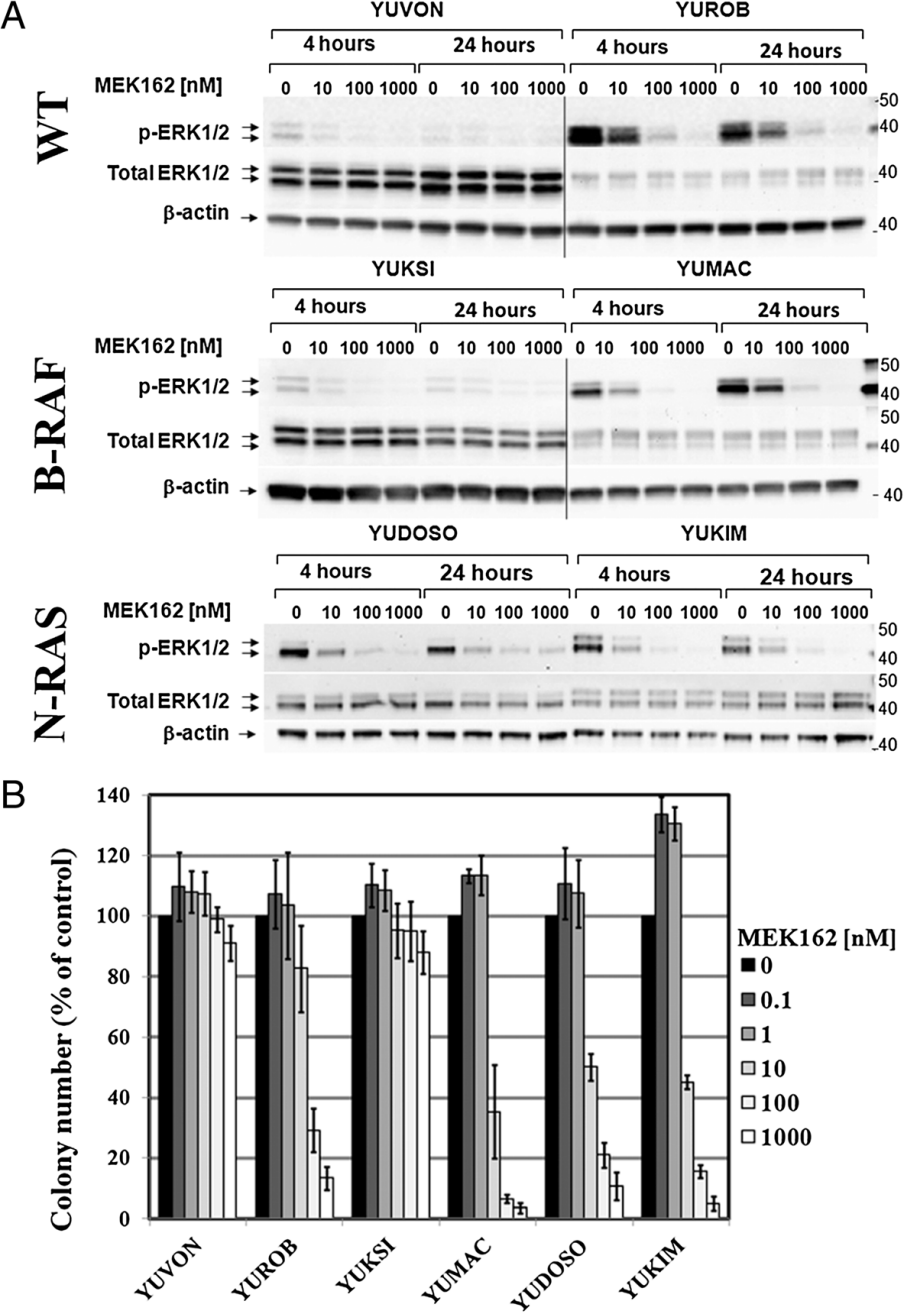
**Effect of MEK162 treatment on ERK1/2 phosphorylation and clonogenic survival of melanoma cells. (A)** WT (YUVON and YUROB), B-RAF mutant (YUKSI and YUMAC) and N-RAS mutant (YUDOSO and YUKIM) cells were treated with increasing doses (10-1000 nM) of MEK162 inhibitor or left untreated for 4 and 24 hours. Western blot analysis was performed using phospho-ERK1/2, total ERK1/2 and β-actin antibodies. **(B)** The panel of six melanoma cultures was treated with increasing concentrations of MEK162. Cells were grown until well-defined colonies were formed. Colonies were visualized by crystal violet staining and counted. Colonies were counted and data are presented as percent of treated cells relative to the untreated cells. Each data point represents a mean of four independent experiments +/- standard error.

Due to the striking sensitivity patterns of MEK162, we conducted additional studies to verify target down-regulation in the sensitive and resistant cultures. ERK1/2 isoforms are the immediate downstream substrates and best studied effectors of dual specificity kinases MEK1/2. To assess the effect of MEK1/2 inhibition on ERK1/2 activation state (phosphorylation at T202/Y204 sites), melanoma cultures were treated with MEK162 and compared with untreated controls. We selected one sensitive and one resistant culture in the WT and B-RAF mutant categories. Seeing that all N-RAS mutant cultures were sensitive to MEK162, we selected two sensitive cultures for these studies. WT (YUVON and YUROB), B-RAF mutant (YUKSI and YUMAC) and N-RAS mutant (YUDOSO and YUKIM) cells were treated with increasing doses (10-1000 nM) of MEK162 or left untreated for 4 and 24 hours. Western blot analysis was performed using phospho-ERK1/2, total ERK1/2 and β-actin antibodies, and results are shown in Figure 2A.In the MEK162 resistant melanoma cultures (YUVON and YUKSI), the baseline level of phospho-ERK1/2 and the ratio of phospho-ERK1/2 to total ERK1/2 was lower compared to sensitive cultures (YUROB, YUMAC, YUDOSO, YUKIM). In MEK162-sensitive melanomas exposure to MEK162 resulted in a significant decrease in the level of ERK1/2 phosphorylation (Figure 2A).

### Clonogenic assays

We next examined the effect of MEK162 on clonogenicity of this panel of six melanoma cultures (Figure 2B). Inhibition of colony formation corresponded well to the viability studies conducted on cells (Table 2). Among the sensitive melanoma cultures, YUROB was somewhat resistant at 10 nM MEK162, retaining 80% clonogenicity of the control level, whereas the ability of other sensitive cultures to form colonies at this concentration of MEK162 dropped below 50% of control (see YUMAC, YUDOSO, and YUKIM). The least inhibition was seen with the MEK162 resistant YUVON and YUKSI cells.

### Induction of apoptosis by MEK162

**Figure 3:**
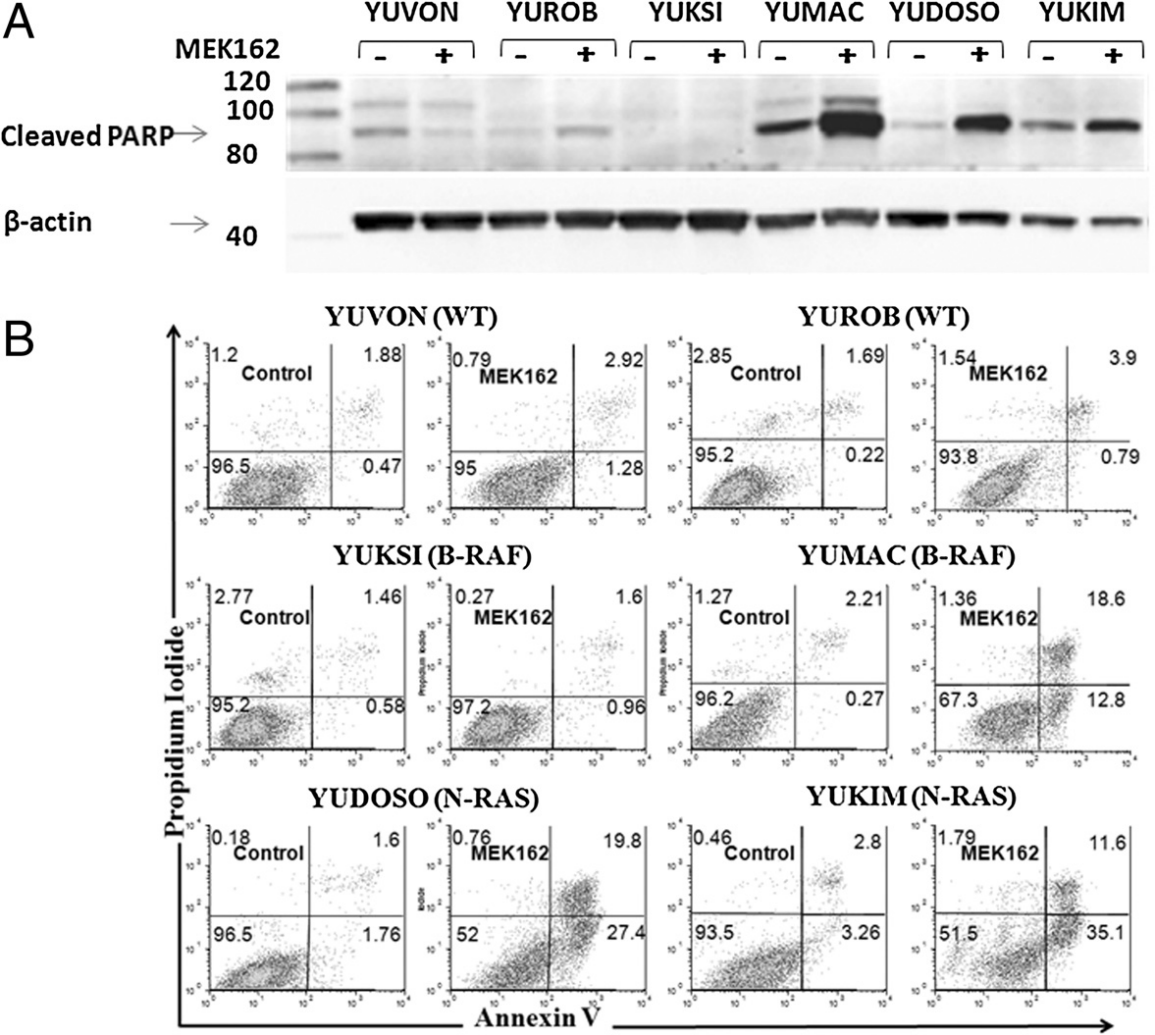
**MEK162 induces apoptosis in drug-sensitive melanoma cells. (A)** A panel of melanoma cultures was treated with MEK162 (1000 nM) or left untreated for 72 hours. Cell lysates were analyzed by western blotting using polyclonal antibody recognizing cleaved PARP. Anti-β-actin western blotting was used as a loading control. **(B)** Cells were treated as in **(A)** and collected for annexin V/propidium iodide labeling followed by FACS analysis. Viable cells are found in the lower left quadrant, apoptotic cells are in the lower right (early apoptotic) and upper right (late apoptotic/necrotic) quadrants; the upper left quadrant represents pyknotic nuclei. Numbers in each quadrant represent the percentage of cells in the corresponding subpopulation.

The MAPK cascade plays a major role in cell survival and proliferation. Hence, MEK162-mediated inhibition of MAPK signaling may result in either cell death, or inhibition of proliferation, or both. Microscopic assessment of sensitive melanoma cell cultures suggested that MEK162 treatment affects cell survival (due to abundance of pyknotic cells). Lysates prepared from MEK162-treated and vehicle-treated cells were resolved by SDS-PAGE and probed with an antibody detecting a cleavage product of a known caspase substrate, PARP (Figure 3A). Cultures were treated with MEK162 (1000 nM) for 72 hours or left untreated. Cell lysates were analyzed by western blotting using an antibody recognizing cleaved PARP. Increased levels of cleaved PARP were seen in the sensitive cultures (Figure 3A). The most abundant PARP cleavage was seen in the sensitive cultures, YUMAC, YUDOSO and YUKIM, and to a much lesser extent in YUROB, whereas no accumulation of cleaved PARP was detected in the resistant cultures (YUVON and YUKSI).

To further examine the inhibitory effect of MEK162 on this panel of melanoma cultures (four MEK162 sensitive: YUROB, YUMAC, YUDOSO, YUKIM, and two MEK162 resistant: YUVON and YUKSI), we assessed apoptosis by annexin V/propidium iodide labeling. Annexin V avidly binds to phosphatidylserine, a phospholipid found exclusively in the intracellular (cytoplasmic) leaflet of the cell membrane under normal physiological conditions but externalized during early steps of apoptotic death. Propidium iodide is used in this assay to differentiate between early and late apoptotic cells, since it cannot penetrate into viable cells possessing an intact membrane. Hence, cells positive for only fluorophore-conjugated annexinV binding represent a dying cell population, whereas doubly stained cells represent a population in late stages of apoptosis. Cells were cultured in the presence or absence of 1000 nM MEK162 for 72 hours, harvested and doubly stained with annexin V/propidium iodide and analyzed by flow cytometry (Figure 3B). The non-apoptotic viable cells are negative for annexin V and propidium iodide staining (bottom left quadrants). Cells at early apoptotic stage show annexin V positive and propidium iodide negative staining (bottom right quadrants), whereas cells at advanced stage of apoptosis are stained positively with annexin V and propidium iodide (upper right quadrants). MEK162 induces robust apoptosis (32-47% of cells were dead or dying) in all sensitive melanoma cultures except YUROB (Figure 3B), consistent with the PARP cleavage data. Resistance of YUROB to MEK162-induced apoptosis may reflect a cytostatic effect since proliferation of this melanoma culture was affected by the drug (Table 2 and Figure 2B).

## Discussion

In this work we studied the clinical characteristics of metastatic melanoma patients whose tumors harbored B-RAF and N-RAS mutations. In our relatively small patient cohort we found a trend towards worse survival and a greater likelihood of brain metastases at the time of initial diagnosis in this patient population. This is consistent with recent interrogation of a larger cohort of patients from MD Anderson Cancer Center in which they showed significantly worse prognosis in this population [27]. We similarly confirmed that patients with B-RAF mutated melanomas are younger than N-RAS mutated counterparts, as previously reported [27,28]. In addition to brain metastases at the time of initial presentation, we found other differences in distribution of metastases in N-RAS mutant melanoma patients, who are more likely to develop metastases to subcutaneous tissues and lymph nodes. Mechanistically, we demonstrate that the MEK inhibitor, MEK162, potently suppresses proliferation of all short term patient-derived N-RAS mutant melanoma cultures tested in our study (n = 7), and this effect is accompanied by robust induction of caspase-dependent apoptosis. Melanoma cultures lacking N-RAS mutation show variable sensitivity to MEK162.

Mutations in B-RAF (50-60%) or N-RAS (15-25%) are frequently found in sun-exposed melanomas and result in hyper-activation of the MAPK pathway [29,30]. Inactivation of oncogenic N-RAS Q61K in N-RAS-driven mouse melanoma model leads to complete tumor regression, implicating N-RAS not only in tumor establishment, but also in tumor maintenance [5]. While mutant B-RAF inhibitors have been successfully developed and approved for the treatment of melanoma, direct targeting of oncogenic RAS isoforms, including N-RAS mutants (Q61K/R/L), is challenging. RAS proteins are small GTPases possessing low catalytic activity. GTP-bound RAS activates signaling cascades via binding and stimulating downstream effectors such as RAF, PI3K, and PLC, whereas the GDP-bound form of RAS is inactive. Under normal physiological conditions RAS activity is strongly dependent on two types of co-factors: stimulatory RAS GEFs (GTP Exchange Factors) and RAS inactivating GAPs (GTPase Activating Proteins). As a result of the oncogenic N-RAS mutations, inefficient RAS GTPase activity is crippled even further, favoring RAS accumulation in the constitutively active, GTP-bound state, resulting in the inability of RAS GAPs to facilitate GTP-hydrolysis [19]. Alternative approaches aimed at de-activating oncogenic RAS indirectly include farnesyl-transferase inhibition and interference with GTP binding [31] or competing out GEF binding [32]. While some of these strategies have already been unsuccessfully tested in clinical trials, others are still being evaluated at the early pre-clinical stage. Thus, although oncogenic RAS isoforms have been found in approximately 33% of all human cancers [33], there are still no drugs that are able to effectively target these oncogenes directly or indirectly.

As there are no drugs that directly target N-RAS, we studied the activity of indirect targeting of downstream effectors; RAF and MEK. Although B-RAF appears to be a plausible target in N-RAS mutated melanoma, preclinical studies have consistently shown that selective B-RAF^V600E^ inhibitors can actually stimulate cell growth and have detrimental effects in N-RAS mutated melanoma [23,24]. One of the mechanisms of the detrimental effects of specific B-RAF targeting in N-RAS mutant melanomas is activation of C-RAF and other downstream mediations. One potential approach to overcome this is pan-RAF inhibition. Our pre-clinical studies, however, using RAF265, suggest that this approach might not be optimal, as only two of the seven N-RAS mutant cultures were sensitive to the drug. RAF265 is no longer in clinical development due to toxicities seen in clinical trials, and other approaches are therefore warranted.

Targeting the N-RAS mutant melanomas with drugs that inhibit signal intermediaries downstream of RAF is an alternative approach. A number of MEK inhibitors are in clinical use or clinical development. Selumetinib, another MEK inhibitor, failed to induce clinical responses in nine melanoma patients whose tumors harbored N-RAS mutations [34]. Trametinib has been used in this population without success; of the nine N-RAS mutant melanoma patients treated on a phase I trial of this drug, none has an objective response [35]. However, objective clinical responses have been seen in over 20% of 28 N-RAS mutant melanoma patients treated with MEK162, and stable disease was seen in additional patients [36]. Due to the small number of samples used in our *in vitro* studies, it is difficult to determine whether MEK162 is superior to trametinib. Very few N-RAS mutant melanoma patients were treated with trametinib and the two drugs have not been compared in a randomized setting. RECIST criteria used in clinical trials require 30% tumor reduction to determine a response, and it is impossible to accurately infer clinical activity from *in vitro* sensitivity data. Additional studies are underway in our laboratory to further explore the RAS/RAF pathway in N-RAS mutant melanomas and determine mechanisms of sensitivity to the various MEK inhibitors.

The clinical activity seen with MEK162 in the earlier phase trial has led to an ongoing phase III randomized trial in this patient population, NCT01763164. Our preclinical findings of remarkable sensitivity to MEK162 in all of seven N-RAS mutant cultures further support this approach. The plasma levels of MEK162 achievable in patients (600-1000 nM) are well above the IC_50_s for N-RAS mutant cultures used in our study (5-13 nM). Furthermore, we demonstrate induction of apoptosis in cultures sensitive to MEK162, suggesting that this drug has cytotoxic effects, in addition to cytostatic effects in N-RAS mutant cells. The importance of these results is underscored by the fact that MEK162 is the first targeted therapy to show clinical activity in patients with N-RAS mutated melanoma. While targeting of mutant B-RAF is possible with such drugs as vemurafenib and dabrafenib, no such targeted therapy is available for patients with N-RAS mutations, who often have aggressive disease requiring rapid anti-tumor intervention, which might be accomplished with targeted therapies.

In conclusion, our data support earlier reports showing that patients with melanomas that harbor oncogenic N-RAS mutations are likely to have shorter overall survival and have brain metastases at the time of initial diagnosis. *In vitro* inhibition of MEK in a panel of short-term melanoma cultures demonstrated exquisite sensitivity in all N-RAS mutant cultures, with resultant induction of apoptosis in sensitive cultures. Although other MEK inhibitors have failed to demonstrate clinical activity in N-RAS mutant melanoma, our findings support further studies of MEK inhibition in this patient population, particularly with MEK162. Given that early phase clinical trials with MEK162 did not show activity in all patients with N-RAS mutant melanomas, predictive biomarker studies are also warranted.

## Materials and methods

### Patient selection and clinical data collection

With approval of a Yale Institutional Review Board, retrospective data were collected from charts of patients treated at the Yale Cancer Center between 2006 and 2010. Only patients for whom B-RAF and N-RAS mutation status was available were included in the analysis. Patient demographics (age, gender), primary tumor characteristics (depth, ulceration, anatomic location), and characteristics at the time of stage IV diagnosis (age, involved sites, serum lactate dehydrogenase [LDH]) were collected. Staging was determined according to the American Joint Committee on Cancer (AJCC) Cancer Staging Manual seventh edition criteria.

### Human melanoma cultures

A panel of 22 low-passage, patient-derived melanoma cultures were obtained from the tissue specimen core of the Yale SPORE in Skin Cancer. Associated patient information is provided in Table 2. Melanoma cultures were maintained in OptiMEM media (Invitrogen, Carlsbad, CA) supplemented with 10% heat-inactivated FBS (Invitrogen) and antibiotic-antimycotic (penicillin, streptomycin, amphotericin B; Invitrogen). Cells were cultured at 37°C in a humidified atmosphere of 95% air/5% CO_2_.

### Cell viability assays

For cell viability assays, cells were plated in triplicate in a 96 well microtiter plate (BD Bioscience) and allowed to grow for 24 hours to an approximate confluence of 30%. MEK162 and and RAF265 were provided as a gift by Novartis Pharmaceuticals. Trametinib was purchased from Selleckchem (Houston, TX). For drug inhibition studies RAF265, MEK162 and trametinib were used to treat melanoma cells at various concentrations. Cell viability was evaluated at 72 hours using the CellTiter-Glo™ Luminescent Cell Viability Assay, according to the manufacturer’s instructions (Promega, USA) and luminescence was measured using a Victor™ X multilabel plate reader (Perkin Elmer). The IC_50_ values were determined by the XLfit software (MathIQ version 2.2.2, IDBS Inc). Experiments were conducted three times and the results represent the average from these independent experiments.

### Western blotting and antibodies

To assess the effect of MEK1/2 inhibition on phospho-ERK1/2 or PARP cleavage, melanoma cells were treated with MEK162 or DMSO. Cells were lyzed in RIPA buffer supplemented with phosphatase and protease inhibitors and protein concentration was determined using Bradford reagent (BioRad Laboratories, Hercules, CA). Protein samples were boiled in Laemli buffer, resolved using 4-20% gradient Criterion™ XT precast gels (BioRad Laboratories) and blotted onto nitrocellulose membranes. To detect phospho-ERK1/2 levels, membranes were probed with rabbit polyclonal phospho-ERK1/2 T202/Y204 antibodies and reprobed with mouse monoclonal antibody recognizing total ERK1/2 (Cell Signaling Technology, Danvers, MA). Rabbit polyclonal antibody raised against cleaved PARP was used to detect the 89 kDa PARP cleavage product (Cell Signaling Technology). For loading control, membranes were stripped in Restore™ Western Blot Stripping Buffer (Thermo Scientific/Pierce, Rockford, IL) and reprobed using anti-β-actin mouse monoclonal antibody (Sigma-Aldrich Corp, St. Louis, MO). Representative results are shown.

### Clonogenic survival assays

YUVON, YUROB, YUKSI, YUMAC, YUDOSO and YUKIM cells were plated at 1,000 per well in six-well plates to provide an optimal counting density. Cells were treated with increasing concentrations of MEK162 (0.1 nM – 1000 nM) and cultured for one to two weeks until well-defined colonies had formed (≥30 cells/colony), replacing culture medium every three days. Cells were fixed and stained with 0.25% w/v crystal violet in 80% methanol solution. Digital images of six-well plates were captured, and colonies were counted using ProtoCOL software (Synbiosis Inc, UK). Data points are an average of four independent experiments and error bars represent the standard error of mean (SEM).

### Annexin V and propidium iodide labeling

Apoptosis in MEK162-treateded melanoma cells was measured using annexin V-Alexa Fluor 488 conjugate apoptosis kit (Invitrogen) according to the manufacturer's instructions. Flow cytometry was performed with a FACScalibur (BD Biosciences), and results were analyzed with FlowJo software (Tree Star, Inc). Experiments were conducted twice independently with similar results. Results of one of the experiments are shown in Figure 3.

### Statistical methods

JMP version 5.0 software was used (SAS Institute, Cary, NC) to analyze the clinical data. Prognostic significance of parameters was assessed using the Cox proportional hazards methods and survival curves were generated using the Kaplan-Meier method. Associations between clinical/pathological parameters and mutational status were assessed by analysis of variance and the Chi square test (*χ*^2^).

## Competing interests

The authors have no potential conflicts of interest to disclose.

## Authors’ contributions

JT and DS performed the bulk of the experiments and data analysis and drafted the manuscript; SAA performed and analyzed cytotoxic assays; JBL performed statistical analysis on patient data and assisted in design of the study, HMK conceived the study, participated in its design and finalized the manuscript. All authors read and approved the final manuscript.

## Supplementary Material

Additional file 1 Patient-derived melanoma cultures with their B-RAF/N-RAS mutational status and sensitivity to MEK162 and trametinib. Click here for file
