# Supplementary material for: MEK targeting in N-RAS mutated metastatic melanoma
Source: Mol Cancer. 2014 Mar 4;13:45. doi: 10.1186/1476-4598-13-45 (PMC3945937; doi:10.1186/1476-4598-13-45)
Supplement: Additional file 1 — Patient-derived melanoma cultures with their B-RAF/N-RAS mutational status and sensitivity to MEK162 and trametinib. [file 1476-4598-13-45-S1.doc]

Supplemental Table 1

|  | **Name of the**  **culture** | **Mutation** | **MEK162 IC_50_s [nM]** | **Trametinib**  **IC_50_s [nM]** |
| --- | --- | --- | --- | --- |
|  |  |  |  |  |
| **Wild type**  **B-RAF /N-RAS** | YUHOIN | WT | >1000 | >1000 |
|  | YUROB | WT | 10 | >1000 |
|  | YUROL | WT | >1000 | >1000 |
|  | YUSOC | WT | 36 | 902 |
|  | YUVON | WT | >1000 | 319 |
| **B-RAF mutants** | YUCOT | V600E (GAG/GAG) | <1 | 374 |
|  | YUGEN | V600E (GAG/GAG) | 108 | 5 |
|  | YUKOLI | V600E/WT (GAG/GTG) | 27 | >1000 |
|  | YUKSI | V600K (AAG/AAG) | 150 | >1000 |
|  | YUMAC | V600K (AAG/AAG) | 8 | 3 |
|  | YURIF | V600K (AAG/AAG) | 45 | 1 |
|  | YUSAC | V600E (GAG/GAG) | 148 | 37 |
|  | YUSIT | V600K/WT (AAG/GTG) | 25 | 118 |
|  | YUSUBA | V600E (GAG/GAG) | 50 | <1 |
|  | YUZEAL | V600R (AGG/AGG) | 33 | <1 |
| **N-RAS mutants** | YUCHER | Q61R (CGA/CGA) | 6 | 20 |
|  | YUDOSO | Q61K/WT (AAA/CAA) | 9 | 1 |
|  | YUFIC | Q61R/WT (CGA/CAA) | 5 | 599 |
|  | YUGANK | Q61K (AAA/AAA) | 5 | 11 |
|  | YUGASP | Q61L (CTA/CTA) | 10 | 1 |
|  | YUKIM | Q61R (AGA/AGA) | 8 | 7 |
|  | YUTICA | Q61R/WT (CGA/CAA) | 13 | 110 |
